# Trends in hypnotic drug use in depression 2007–2017: A Swedish population‐based study

**DOI:** 10.1111/jsr.14267

**Published:** 2024-06-14

**Authors:** Adam Nygren, P. Brenner, L. Brandt, P. Karlsson, S. Eloranta, J. Reutfors

**Affiliations:** ^1^ Centre for Pharmacoepidemiology Karolinska Institutet Stockholm Sweden; ^2^ Centre for Psychiatry Research, Department of Clinical Neuroscience Karolinska Institutet, & Stockholm Health Care Services Stockholm Sweden; ^3^ Division of Clinical Epidemiology Karolinska Institutet Stockholm Sweden

**Keywords:** benzodiazepines, depression, drug utilisation, hypnotic and sedatives, melatonin, Z‐drugs

## Abstract

Insomnia is a common feature of depression; however, depression treatment guidelines provide limited recommendations regarding hypnotic drugs. Few studies have thoroughly investigated the use of hypnotic drugs in depression. In this cohort study using national Swedish registers, we included all patients ≥18 years with incident unipolar depression during 2007–2017. Patients were followed for 3 years, noting the annual and quarterly prevalence of hypnotic drug use from prescription fills. Prevalence ratios (PR) comparing 2017 to 2007 were calculated with 95% confidence intervals (CI). A total of 222,077 patients with depression were included (mean age 41 years, 59% women). In the year following diagnosis, 44.1% used any hypnotic drug in 2017, compared with 46.7% in 2007 (PR 0.94, 95% CI 0.92–0.97). The most commonly used drugs were Z‐drugs (zopiclone, zolpidem, and zaleplon) with a prevalence of 27.6% in 2017 and 35.6% in 2007 (PR 0.78, 95% CI 0.75–0.80). Melatonin use increased sharply to 12.0% in 2017 from 0.4% in 2007 (PR 28.9, 95% CI 23.5–35.7). Hypnotic drug use was most prevalent in the first two quarters after diagnosis; however, after 3 years, the quarterly prevalence was still 19.2%. Hypnotics were more common among women, older patients, those with somatic comorbidities, more severe depression, or a history of suicide attempt. Evidence from this large register‐based study demonstrates that hypnotics were used to a large extent in depression in Sweden 2007–2017. Z‐drugs use declined and melatonin use increased dramatically. Hypnotic drug use remained high even 3 years after diagnosis.

## 1 INTRODUCTION

Around 80% of patients with depression report symptoms of insomnia (Ohayon, 2002; Sunderajan et al., 2010). Conversely, about 25% of patients with symptoms of insomnia also report symptoms of depression (Oh et al., 2019). Insomnia is also associated with a higher risk of later developing depression, with the relative risk found to be approximately doubled (Baglioni et al., 2011; Hertenstein et al., 2019; Li et al., 2016) and is associated with more severe symptoms in depression. Insomnia symptoms may persist even after remission in depression (Fang et al., 2019; McClintock et al., 2011), which increases the risk of depression recurrence (Sakurai et al., 2017). At present, insomnia is viewed as intimately and bidirectionally connected with depression through common mechanisms (Johansson et al., 2021; Riemann et al., 2020; Sivertsen et al., 2012).

Research on the effectiveness of hypnotics in insomnia primarily focusses on benzodiazepine hypnotics and the benzodiazepine‐related “Z‐drugs” zolpidem, zaleplon, zopiclone, and eszopiclone. However, these drugs are recommended for short‐term use only, due to well‐established risks for tolerance and addiction, falls and fractures, motor vehicle accidents, and cognitive side‐effects (Riemann et al., 2023). In Sweden, the benzodiazepine hypnotics nitrazepam and flunitrazepam were previously indicated for insomnia, but were deregistered in 2019 and 2020, respectively. The use of benzodiazepines and related drugs has declined in all Nordic countries from 2010 to 2020, likely as result of guideline changes and other initiatives (Højlund et al., 2023). Another class of hypnotic drugs is antihistamines. Propiomazine (trade name Propavan®), a phenothiazine derivate with antihistaminergic properties, is indicated for insomnia in Sweden and is structurally related to promethazine, among others (Morton & Hall, 2012). Melatonin is also used as a hypnotic drug, although it is often not recommended in guidelines for the treatment of insomnia (Sateia et al., 2017). The Swedish Dental and Pharmaceutical Benefits Agency decided in 2015 to reimburse melatonin, and in 2016 to remove the requirement of a special license for prescription.

Long‐term pharmacological treatments remain common in chronic insomnia (Bertisch et al., 2014), although various guidelines now recommend cognitive behavioural therapy for insomnia (CBT‐I) as the first‐line treatment (Qaseem, Kansagara, et al., 2016; Riemann et al., 2023). CBT‐I has been shown to alleviate both insomnia and depression in patients with both conditions (Hertenstein et al., 2022). Most guidelines on the treatment of depression do not address the pharmacological treatment of concurrent sleep disturbances in detail (Bennabi et al., 2019; Kennedy et al., 2016; National Institute for Health and Care Excellence, 2022; Qaseem, Barry, & Kansagara, 2016). The depression treatment guidelines of the American Psychiatric Association, however, mention that adjunctive hypnotic drug treatment may hasten symptomatic relief but that there is no evidence of long‐term benefit, and that tapering may be difficult (American Psychiatric Association, 2010). Similarly, guidelines from The Royal Australian and New Zealand College of Psychiatrists (Malhi et al., 2021) mention that the use of hypnotics is acceptable only for short‐term sleep problems.

Some previous studies have addressed the utilisation of hypnotic drugs in depression. In a Finnish sample of 288 patients with depression interviewed during 2000 to 2001, 16% used sedatives/hypnotics (Hämäläinen et al., 2009). In a secondary analysis of the STAR*D study including more than 3700 patients, 84.7% had insomnia symptoms, while only 27% were taking hypnotics at study entry, which the authors suggested could indicate under‐treatment (Sunderajan et al., 2010). In a Swedish study of depression treatment in patients over 60 years old without dementia, 41% of 182 patients with depression (minor or major) used hypnotics (Karlsson et al., 2016). However, most previous studies did not specify the use of different drug classes or in subgroups of patients with depression, and none of them were population‐based studies covering all adult patients with incident depression.

The aim of this study was to assess the use of hypnotics in depression in the Swedish setting using national registers with near‐complete coverage, and to study the use over time.

## 2 METHODS

### 2.1 Data sources

We used data from Swedish health registers, which were linked using the unique personal identification number assigned to each Swedish citizen at birth or immigration. Data on psychiatric inpatient and outpatient treatment were obtained from the National Patient Register (NPR), which has been in use since 1964 and provides complete national coverage of inpatient care since 1987. Since 2001, similar information has also been collected on outpatient visits in specialised care. Diagnoses are coded according to the International Statistical Classification of Diseases and Related Health Problems (ICD), with version 10 used since 1997. The Prescribed Drug Register (PDR) provided data on filled prescriptions of psychotropic drugs. This register contains information on all dispensed drugs at Swedish pharmacies since July 2005 but does not include drugs administered in hospitals (Wettermark et al., 2007). Data on marital status and education were linked from national registers held at Statistics Sweden.

### 2.2 Study population

All patients in Sweden at least 18 years of age with an incident diagnosis of a unipolar depressive episode (ICD‐10 code F32–F33) recorded as a primary diagnosis in psychiatric specialist care (inpatient or outpatient) during 2007–2019 were identified in the NPR. Incident cases were defined as those without any recorded depression diagnosis in psychiatric specialist care during the 5 years prior to the depression diagnosis. The date of the incident depression diagnosis was considered the index date. There was no requirement of a diagnosis of insomnia. We excluded patients with a diagnosis of psychosis (ICD‐10 F20–F29), mania (F30), bipolar disorder (F31), or dementia/organic brain disorder (F00–09) in the NPR during the 5 years before and including the index date. Additionally, patients who had filled a prescription for any antipsychotic (Anatomical Therapeutic Chemical [ATC] code N05A) or mood stabiliser treatment (N03AG01, N03AX09, N03AF01) within 1 year before inclusion were excluded. The same patient could be included more than once, provided that at least 5 years had passed since the last depression diagnosis.

### 2.3 Outcomes and covariates

All dispensed prescriptions of drugs licensed in Sweden for the treatment of insomnia were identified in the PDR during the year (365 days) after the index date. This included Z‐drugs (N05CF), propiomazine (N05CM06), melatonin (N05CH01), and benzodiazepine hypnotics (nitrazepam N05CD02 and flunitrazepam N05CD03). To study long‐term use, fills of these drugs were also examined in quarterly intervals over a 3‐year period after the index date.

The following patient characteristics were recorded when entering the study for each patient when entering the study: age, sex, marital status, highest educational level, and country of birth. The depressive episode was categorised as “severe depression” when the ICD‐10 codes F32.2–3 or F33.2–3 were used for the index diagnosis or had appeared within 30 days thereafter. A history of suicide attempt was identified as ICD‐10 codes X60–84 or Y10–34 in the NPR at any time before and including the index date. Additionally, we identified any diagnosis of anxiety disorders (ICD‐10 code F40–F48), personality disorders (F60–F61), and substance use disorders, during 5 years before and including the index date. Substance use disorders were categorised as alcohol use disorder only (F10.1–9 and/or prescription of disulfiram [N07BB01], acamprosate [N07BB03], naltrexone [N07BB04], or nalmefene [N07BB05]), or other/combined (F11–16 and/or F18–19, and/or prescriptions of sublingual buprenorphine [N07BC01/N07BC51] or methadone [N07BC02]). The Charlson comorbidity index (Charlson et al., 1987) was used to estimate the burden of somatic diseases of the patients categorised as 0, 1–2, or ≥3 major comorbidities based on the number of registered diagnoses in the NPR during the 5 years before and including the index date.

### 2.4 Statistical analyses

The one‐year (annual) prevalence of hypnotic drug use was calculated by dividing the total number of patients who had filled at least one prescription for each drug class/specific drug by the number of patients with a diagnosis of depression during each year of the study period. Changes in temporal trends were assessed using prevalence ratios (PR) together with 95% confidence intervals (CI), calculated by dividing the prevalence in 2017 by that in 2007. The prevalence of hypnotic drug use by drug class/specific drug was also presented graphically, with time since the first registered depression diagnosis on the x‐axis.

### 2.5 Ethical permission

The study was approved by the regional ethical review board in Stockholm (no. 2017/1236–31/2).

## 3 RESULTS

Characteristics of the study population are presented in Table 1. A total of 222,077 patients with depression were included in the study (mean age 41 (SD ±17) years, 59% women, 87% diagnosed in outpatient care). A history of suicide attempt was recorded among 8.5%. Anxiety disorder was the most common comorbid psychiatric diagnosis, found among 32.5% of the patients, and 13.9% had a severe depressive episode.

**TABLE 1:** Characteristics of patients with incident diagnosis of depression in Sweden 2007–2017.

	*N* (%)
All patients	222,077 (100)
Men	91,051 (41.0)
Women	131,026 (59.0)
Age (years) at diagnosis	
18–29	73,151 (32.9)
30–49	83,607 (37.6)
50–69	48,095 (21.7)
≥ 70	17,224 (7.8)
Marital status	
Unmarried	159,433 (71.8)
Married	612,78 (27.6)
Missing	1366 (0.6)
Education	
Low (≤9 years)	54,869 (24.7)
Middle (10–12 years)	100,664 (45.3)
High (≥13 years)	62,069 (27.9)
Missing	4475 (2.0)
Index diagnosis	
Inpatient care	28,709 (12.9)
Outpatient care	193,368 (87.1)
Severe/psychotic depression^a^	30,880 (13.9)
Suicide attempt^b^, ^c^	18,969 (8.5)
Anxiety disorder^d^, ^e^	72,139 (32.5)
Personality disorder^d^, ^f^	5436 (2.4)
ADHD^d^, ^g^	7285 (3.3)
Substance use disorder^d^	
Alcohol use disorder^h^	15,404 (6.9)
Other drugs or mixed^i^	10,001 (4.5)
Charlson Comorbidity Index^d^	
0	196,534 (88.5)
1–2	24,179 (10.9)
≥ 3	1364 (0.6)

^a^ Defined as any registered ICD‐10 code of F32.2–3 or F33.2–3 at or within first 30 days after index depression diagnosis.

^b^ At any time before and including date of index diagnosis.

^c^ ICD‐10 code X60–84 or Y10–34.

^d^ During 5 years before and including date of index diagnosis.

^e^ ICD‐10 code F40 − F48.

^f^ ICD‐10 code F60−F61.

^g^ ICD‐10 code F90.

^h^ ICD‐10 code F10.1–9 and/or prescription fill of disulfiram (N07BB01), acamprosate (N07BB03), naltrexone (N07BB04), or nalmefene (N07BB05), and no other drugs.

^i^ ICD‐10 code F11–16 and/or F18–9 and/or prescription fill of sublingual buprenorphine (N07BC01/ N07BC51) or methadone (N07BC02).

**FIGURE 1:**
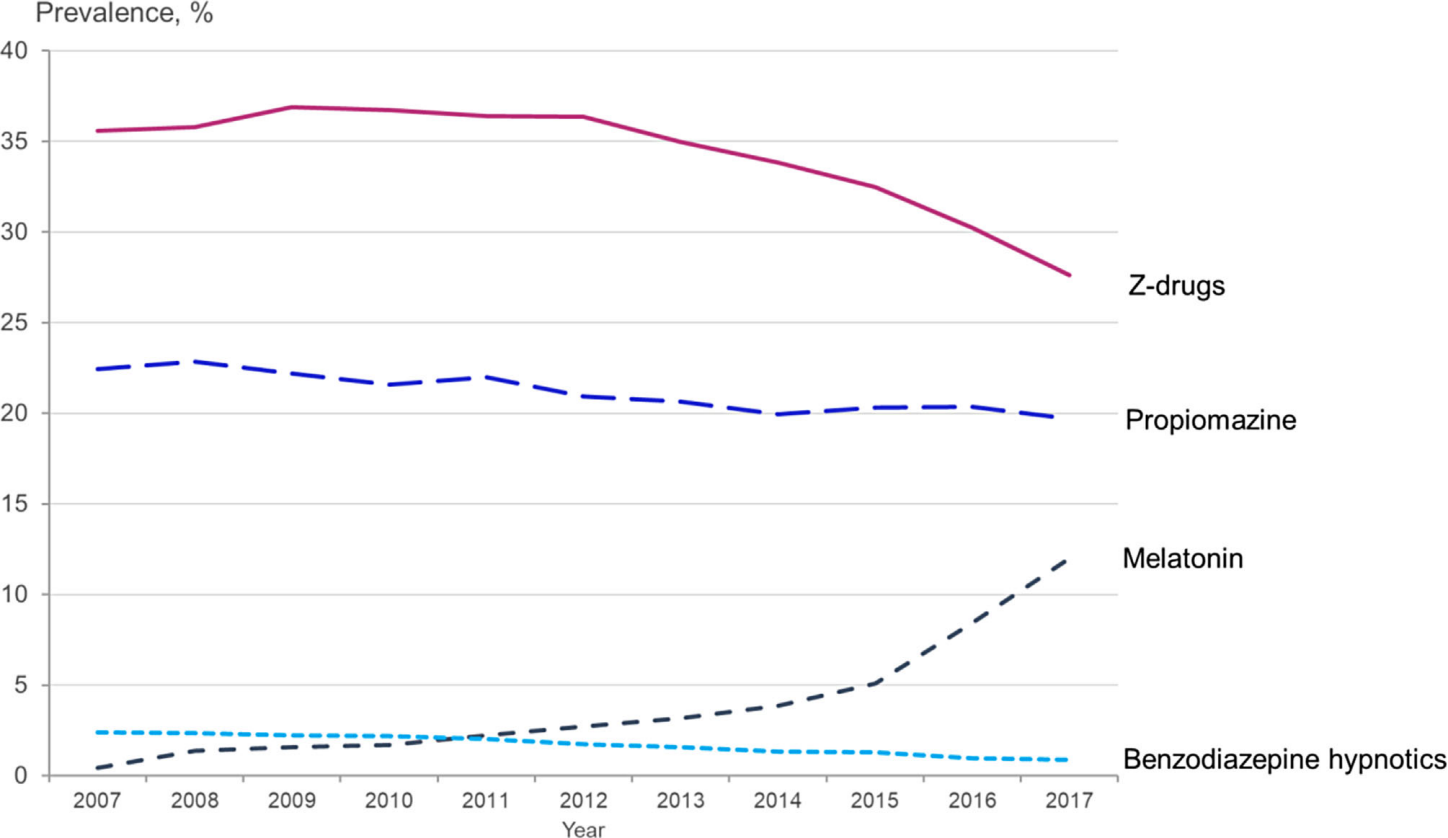
Trends in annual prevalence of hypnotic drug use among patients with incident depression in Sweden 2007–2017.

The overall hypnotic drug use in depression is depicted in Figure 1, with a detailed comparison between 2007 and 2017 provided in Table 2. The results show that nearly half of the patients filled a prescription for a hypnotic drug in the year following their depression diagnosis, with a slight decrease from 46.7% in 2007 to 44.1% in 2017 (PR 0.94, 95% CI 0.92–0.97). Z‐drugs were the most common hypnotic drugs used by 35.6% of patients in 2007 and 27.6% in 2017 (PR 0.78, 95% CI 0.75–0.80). Among the Z‐drugs, zopiclone was the most frequently used, although its proportion of users decreased from 24.9% to 22.9% (PR 0.92, 95% CI 0.89–0.95), while zolpidem use more than halved between 2007 and 2017. The use of zaleplon was negligible. The proportion of users of benzodiazepine drugs indicated for insomnia was low and decreased significantly from 2007 to 2017: flunitrazepam from 1.6% to 0.6% (PR 0.39, 95% CI 0.32–0.49), and nitrazepam from 0.8% to 0.2% (PR 0.32, 95% CI 0.23–0.44). The use of the phenothiazine drug propiomazine was the second highest after Z‐drugs, and decreased moderately from 22.4% in 2007 to 19.7% in 2017 (PR 0.88, 95% CI 0.85–0.91). The most significant change in medication use over the study period was observed for melatonin, as the proportion of users increased nearly 30‐fold from 0.4% in 2007 to 12.0% in 2017 (PR 28.9, 95% CI 23.5–35.7).

**TABLE 2:** Annual prevalence of hypnotic drug use in the year after an incident diagnosis of depression in Sweden in 2007 (*N* = 21,754) and 2017 (*N* = 18,011)

	All patients	Women	Men
Any hypnotic drug			
2007, *N* (%)	10,164 (46.7)	6216 (46.9)	3948 (46.4)
2017, *N* (%)	7952 (44.2)	4688 (45.2)	3264 (42.7)
PR (95% CI)	0.94 (0.92–0.97)	0.96 (0.94–0.99)	0.92 (0.89–0.95)
Z‐drugs			
2007, *N* (%)	7745 (35.6)	4822 (36.4)	2923 (34.4)
2017, *N* (%)	4973 (27.6)	3010 (29.0)	1963 (25.7)
PR (95% CI)	0.78 (0.75–0.80)	0.80 (0.77–0.83)	0.75 (0.71–0.78)
Zopiclone			
2007, *N* (%)	5420 (24.9)	3296 (24.9)	2124 (25.0)
2017, *N* (%)	4118 (22.9)	2464 (23.8)	1654 (21.6)
PR (95% CI)	0.92 (0.89–0.95)	0.96 (0.91–1.00)	0.87 (0.82–0.92)
Zolpidem			
2007, *N* (%)	3182 (14.6)	2084 (15.7)	1098 (12.9)
2017, *N* (%)	1256 (7.0)	803 (7.7)	453 (5.9)
PR (95% CI)	0.48 (0.45–0.51)	0.49 (0.46–0.53)	0.46 (0.41–0.51)
Zaleplone			
2007, *N* (%)	145 (0.7)	91 (0.7)	54 (0.6)
2017, *N* (%)	0	0	0
PR (95% CI)	‐	‐	‐
Benzodiazepine hypnotics			
2007, *N* (%)	517 (2.4)	301 (2.3)	216 (2.5)
2017, *N* (%)	156 (0.9)	95 (0.9)	61 (0.8)
PR (95% CI)	0.36 (0.31–0.44)	0.40 (0.32–0.51)	0.31 (0.24–0.42)
Flunitrazepam			
2007, *N* (%)	355 (1.6)	202 (1.5)	153 (1.8)
2017, *N* (%)	116 (0.6)	72 (0.7)	44 (0.6)
PR (95% CI)	0.39 (0.32–0.49)	0.46 (0.35–0.60)	0.32 (0.23–0.45)
Nitrazepam			
2007, *N* (%)	178 (0.8)	107 (0.8)	71 (0.8)
2017, *N* (%)	47 (0.3)	29 (0.3)	18 (0.2)
PR (95% CI)	0.32 (0.23–0.44)	0.35 (0.23–0.52)	0.28 (0.17–0.47)
Propiomazine			
2007, *N* (%)	4879 (22.4)	2919 (22.0)	1960 (23.1)
2017, *N* (%)	3550 (19.7)	2033 (19.6)	1517 (19.8)
PR (95% CI)	0.88 (0.85–0.91)	0.89 (0.85–0.94)	0.86 (0.81–0.91)
Melatonin			
2007, *N* (%)	90 (0.4)	53 (0.4)	37 (0.4)
2017, *N* (%)	2157 (12.0)	1281 (12.4)	876 (11.5)
PR (95% CI)	28.95 (23.47–35.71)	30.89 (23.50–40.61)	26.34 (18.98–36.54)

Abbreviations: CI, confidence interval; PR, prevalence ratio comparing 2017 to 2007.

When analysing the data separately for women and men, the overall use of hypnotic drugs decreased slightly more among men (PR 0.92, 95% CI 0.89–0.95) compared with women (PR 0.96, 0.94–0.99) (Table 2). This difference was primarily due to the decreased use of Z‐drugs, with a decrease from 34% to 25% among men and from 36% to 29% among women between 2007 and 2017. Conversely, the increase in melatonin use was higher in women (PR 30.9, 95% 23.5–40.6) than in men (PR 26.3, 95% CI 19.0–36.5). The overall trends are also presented in Figure 1.

The use of hypnotic drugs in relation to sociodemographic and clinical characteristics in 2007 and 2017 is presented in Table 3. The highest use was observed among patients over 70 years old, with 59.2% of them filling a prescription for any hypnotic drug in 2017. Married patients and those with shorter education had higher rates of hypnotic drug use. A higher proportion of those diagnosed in hospital (58.3%) used hypnotics compared with those diagnosed as outpatients (42.9%). Furthermore, patients with severe depression or a history of suicide attempt had a higher prevalence of hypnotic use. Among patients with comorbid psychiatric diagnoses, those with substance use disorders (non‐alcohol/mixed) had the highest hypnotic drug use. The highest use was found in patients with a greater burden of somatic comorbidity, with 60.3% of those with a Charlson comorbidity index score of 3 or more using any hypnotic drug.

**TABLE 3:** Annual prevalence of hypnotic drug use in the year after an incident diagnosis of depression in 2007 (*N* = 21,754) and 2017 (*N* = 18,011) by sociodemographic and clinical characteristics.

	Any hypnotic drug			Z‐drugs			Benzodiazepine hypnotics			Propiomazine			Melatonin		
	2007	2017	PR	2007	2017	PR	2007	2017	PR	2007	2017	PR	2007	2017	PR
	*N* (%)	*N* (%)	(95% CI)	*N* (%)	*N* (%)	(95% CI)	*N* (%)	*N* (%)	(95% CI)	*N* (%)	*N* (%)	(95% CI)	*N* (%)	*N* (%)	(95% CI)
**Age (years)**															
18–29	2200 (36.4)	2540 (36.5)	1.00 (0.96–1.05)	1551 (25.7)	1133 (16.3)	0.64 (0.59–0.68)	58 (1.0)	17 (0.2)	0.25 (0.15–0.44)	1204 (19.9)	1230 (17.7)	0.89 (0.83–0.95)	25 (0.4)	966 (13.9)	33.59 (22.62–49.89)
30–49	3998 (46.1)	2747 (44.0)	0.96 (0.92–0.99)	3012 (34.7)	1718 (27.5)	0.79 (0.75–0.83)	173 (2.0)	60 (1.0)	0.48 (0.36–0.65)	2020 (23.3)	1331 (21.3)	0.92 (0.86–0.97)	23 (0.3)	724 (11.6)	43.77 (28.93–66.20)
50–69	2970 (56.4)	1826 (53.7)	0.95 (0.92–0.99)	2351 (44.6)	1369 (40.3)	0.90 (0.86–0.95)	187 (3.5)	46 (1.4)	0.38 (0.28–0.52)	1335 (25.3)	798 (23.5)	0.93 (0.86–1.00)	32 (0.6)	339 (10.0)	16.41 (11.45–23.52)
≥70	996 (56.4)	839 (59.2)	1.05 (0.99–1.11)	831 (47.1)	753 (53.1)	1.13 (1.05–1.21)	99 (5.6)	33 (2.3)	0.42 (0.28–0.61)	320 (18.1)	191 (13.5)	0.74 (0.63–0.88)	10 (0.6)	128 (9.0)	15.94 (8.41–30.23)
Unknown	51 (44.0)	41 (30.6)	0.70 (0.50–0.97)	40 (34.5)	21 (15.7)	0.45 (0.29–0.72)	3 (2.6)	1 (0.7)	0.29 (0.03–2.74)	26 (22.4)	24 (17.9)	0.80 (0.49–1.31)	‐	6 (4.5)	‐
**Marital status**															
Unmarried	6690 (44.6)	5770 (42.8)	0.96 (0.93–0.98)	5062 (33.8)	3437 (25.5)	0.75 (0.73–0.78)	313 (2.1)	99 (0.7)	0.35 (0.28–0.44)	3252 (21.7)	2614 (19.4)	0.89 (0.85–0.93)	66 (0.4)	1678 (12.4)	28.23 (22.10–36.06)
Married	3423 (51.5)	2141 (48.9)	0.95 (0.91–0.99)	2643 (39.7)	1515 (34.6)	0.87 (0.83–0.92)	201 (3.0)	56 (1.3)	0.42 (0.32–0.57)	1601 (24.1)	912 (20.8)	0.86 (0.80–0.93)	24 (0.4)	473 (10.8)	29.92 (19.89–45.01)
**Education**															
≤9 years	2725 (47.2)	1750 (44.6)	0.94 (0.90–0.99)	2044 (35.4)	1037 (26.4)	0.75 (0.70–0.79)	143 (2.5)	27 (0.7)	0.28 (0.18–0.42)	1298 (22.5)	734 (18.7)	0.83 (0.77–0.90)	21 (0.4)	503 (12.8)	35.24 (22.81–54.42)
10–12 years	4704 (46.7)	3749 (45.9)	0.98 (0.95–1.01)	3570 (35.4)	2356 (28.8)	0.81 (0.78–0.85)	239 (2.4)	76 (0.9)	0.39 (0.30–0.51)	2319 (23.0)	1720 (21.0)	0.91 (0.87–0.97)	41 (0.4)	991 (12.1)	29.81 (21.84–40.68)
≥13 years	2539 (46.6)	2338 (42.2)	0.90 (0.87–0.94)	1987 (36.5)	1517 (27.4)	0.75 (0.71–0.79)	124 (2.3)	51 (0.9)	0.40 (0.29–0.56)	1155 (21.2)	1035 (18.7)	0.88 (0.82–0.95)	28 (0.5)	640 (11.6)	22.46 (15.41–32.73)
**Index diagnosis setting**															
Outpatient	8307 (44.4)	7076 (42.9)	0.96 (0.94–0.99)	6260 (33.5)	4371 (26.5)	0.79 (0.77–0.82)	413 (2.2)	134 (0.8)	0.37 (0.30–0.45)	3925 (21.0)	3099 (18.8)	0.89 (0.86–0.93)	78 (0.4)	1950 (11.8)	28.31 (22.59–35.46)
Inpatient	1857 (60.6)	876 (58.3)	0.96 (0.91–1.01)	1485 (48.5)	602 (40.1)	0.83 (0.77–0.89)	104 (3.4)	22 (1.5)	0.43 (0.27–0.68)	954 (31.1)	451 (30.0)	0.96 (0.88–1.06)	12 (0.4)	207 (13.8)	35.15 (19.71–62.70)
Unknown	196 (42.4)	115 (30.6)	0.72 (0.60–0.87)	144 (31.2)	63 (16.8)	0.54 (0.41–0.70)	11 (2.4)	2 (0.5)	0.22 (0.05–1.00)	107 (23.2)	61 (16.2)	0.70 (0.53–0.93)	‐	23 (6.1)	‐
**Severe/psychotic depression** ^a^	1793 (55.0)	1262 (54.8)	1.00 (0.95–1.04)	1450 (44.5)	859 (37.3)	0.84 (0.78–0.89)	108 (3.3)	33 (1.4)	0.43 (0.29–0.64)	845 (25.9)	609 (26.4)	1.02 (0.93–1.11)	17 (0.5)	299 (13.0)	24.87 (15.30–40.42)
**Suicide attempt** ^b^, ^c^	895 (53.2)	870 (53.6)	1.01 (0.95–1.07)	695 (41.3)	571 (35.2)	0.85 (0.78–0.93)	38 (2.3)	30 (1.8)	0.82 (0.51–1.31)	462 (27.5)	405 (25.0)	0.91 (0.81–1.02)	6 (0.4)	236 (14.5)	40.79 (18.19–91.45)
**Anxiety disorder** ^d^, ^e^	2720 (49.1)	3261 (46.4)	0.94 (0.91–0.98)	2123 (38.3)	2084 (29.6)	0.77 (0.74–0.81)	143 (2.6)	74 (1.1)	0.41 (0.31–0.54)	1310 (23.6)	1425 (20.3)	0.86 (0.80–0.92)	21 (0.4)	952 (13.5)	35.72 (23.21–54.97)
**Personality disorder** ^d^, ^f^	225 (40.6)	195 (48.3)	1.19 (1.03–1.37)	179 (32.3)	130 (32.2)	1.00 (0.83–1.20)	14 (2.5)	5 (1.2)	0.49 (0.18–1.35)	104 (18.8)	78 (19.3)	1.03 (0.79–1.34)	4 (0.7)	60 (14.9)	20.57 (7.54–56.14)
**ADHD** ^d^, ^g^	62 (36.5)	546 (45.4)	1.24 (1.01–1.53)	51 (30.0)	279 (23.2)	0.77 (0.60–0.99)	3 (1.8)	9 (0.7)	0.42 (0.12–1.55)	26 (15.3)	183 (15.2)	0.99 (0.68–1.45)	1 (0.6)	292 (24.3)	41.26 (5.83–292.0)
**Substance use disorder** ^d^															
Alcohol use disorder^h^	692 (49.3)	517 (45.7)	0.93 (0.85–1.01)	486 (34.6)	304 (26.9)	0.78 (0.69–0.87)	25 (1.8)	10 (0.9)	0.50 (0.24–1.03)	393 (28.0)	267 (23.6)	0.84 (0.74–0.96)	6 (0.4)	122 (10.8)	25.22 (11.16–57.03)
Other or mixed substance use disorder^i^	404 (53.8)	480 (53.0)	0.99 (0.90–1.08)	299 (39.8)	299 (33.0)	0.83 (0.73–0.94)	35 (4.7)	15 (1.7)	0.36 (0.20–0.65)	197 (26.2)	213 (23.5)	0.90 (0.76–1.06)	4 (0.5)	152 (16.8)	31.53 (11.74–84.70)
**Charlson Comorbidity Index** ^d^															
0	8803 (45.6)	6882 (42.8)	0.94 (0.92–0.96)	6628 (34.4)	4136 (25.7)	0.75 (0.72–0.77)	423 (2.2)	122 (0.8)	0.35 (0.28–0.42)	4325 (22.4)	3165 (19.7)	0.88 (0.84–0.91)	79 (0.4)	1964 (12.2)	29.81 (23.83–37.29)
1–2	1300 (55.2)	1000 (55.5)	1.01 (0.95–1.06)	1063 (45.1)	773 (42.9)	0.95 (0.89–1.02)	87 (3.7)	33 (1.8)	0.50 (0.33–0.74)	530 (22.5)	368 (20.4)	0.91 (0.81–1.02)	11 (0.5)	187 (10.4)	22.24 (12.14–40.72)
≥3	61 (59.2)	70 (60.3)	1.02 (0.82–1.27)	54 (52.4)	64 (55.2)	1.05 (0.82–1.35)	7 (6.8)	1 (0.9)	0.13 (0.02–1.01)	24 (23.3)	17 (14.7)	0.63 (0.36–1.10)	‐	6 (5.2)	‐

Abbreviations: CI, confidence interval; PR, prevalence ratio comparing 2017 to 2007.

^a^ Defined as any registered ICD‐10 code of F32.2–3 or F33.2–3 at or within first 30 days after index depression diagnosis.

^b^ At any time before and including date of index diagnosis.

^c^ ICD‐10 code X60–84 or Y10–34.

^d^ During 5 years before and including date of index diagnosis.

^e^ ICD‐10 code F40−F48.

^f^ ICD‐10 code F60−F61.

^g^ ICD‐10 code F90.

^h^ ICD‐10 code F10.1–9 and/or prescription fill of disulfiram (N07BB01), acamprosate (N07BB03), naltrexone (N07BB04), or nalmefene (N07BB05), and no other drugs.

^i^ ICD‐10 codes F11–16, and/or F18–9 and/or prescription fill of sublingual buprenorphine (N07BC01/N07BC51) or methadone (N07BC02).

Z‐drugs were most commonly used among patients aged 70 years or older (53.1%), with the same groups that had the highest overall hypnotic drug use also having the highest use of Z‐drugs. On the other hand, melatonin use was highest among younger patients (13.9% in the 18–29 age group), those with an ADHD diagnosis (24.3%), and those who were relatively physically healthy.

Comparing the use in 2007 to 2017, Z‐drugs decreased in all age groups except the oldest, where an increase was observed (PR 1.13, 95% CI 1.05–1.21) (Table 3). Benzodiazepine hypnotics showed a marked decrease in all age groups. The decrease in propiomazine use was relatively small, but more evident in the highest age group. The most notable increase was seen in melatonin use, especially in the youngest age groups below 50 years, and among those with comorbid ADHD.

**FIGURE 2:**
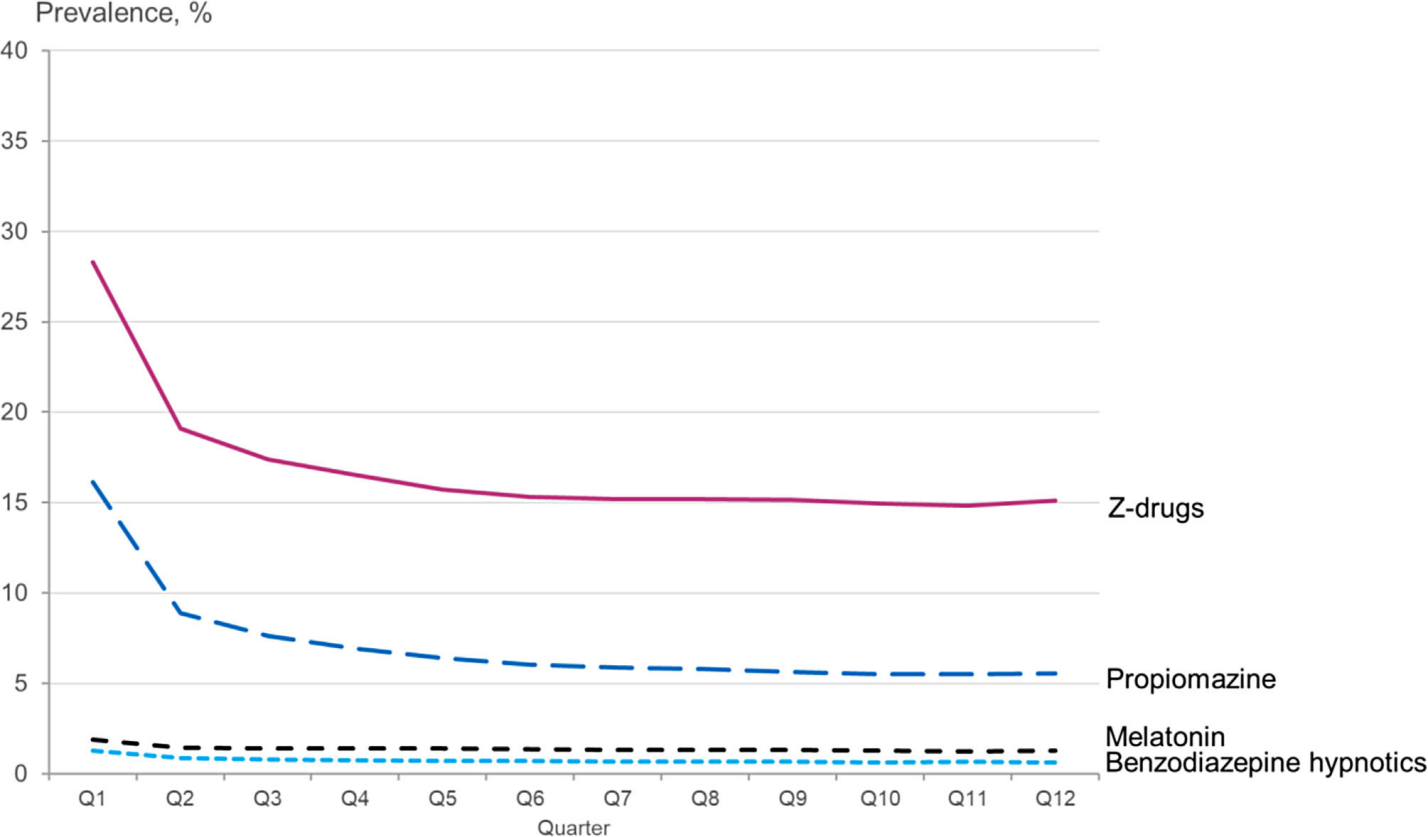
Prevalence of hypnotic drug use among patients with incident depression at each quarter year (Q) since depression diagnosis.

Examining the trends of hypnotic drug use in the first 3 years after the depression diagnosis the highest use was observed in the first two 90‐day periods (Figure 2). Drug use continued to decrease during the following 360 days; however, after six 90‐day periods, the proportions of users remained stable, indicating persistence in drug use. After 3 years, 19.2% of patients were still using at least one hypnotic drug.

## 4 DISCUSSION

In this nationwide population‐based study of patients with newly diagnosed depression, we observed that almost half of the patients used hypnotic drugs at some point in the year following their diagnosis. Even 4 years after diagnosis, up to 20% of patients continued to use hypnotic drugs. Patients with more severe depression or somatic comorbidity exhibited higher levels of hypnotic drug use. From 2007 to 2017, the use of all hypnotic drug classes decreased in use, as a proportion of all patients with depression, except for melatonin, which showed an almost 30‐fold increase.

This was a large cohort study and patients were followed in registers with minimal attrition. The validity of the National Patient Register's psychiatric diagnoses has been found to be high (Ludvigsson et al., 2011). Similarly, the quality of data in the Prescribed Drug Register is high (The National Board of Health and Welfare, 2022), but with the limitation of not including drugs given in hospitals. Another limitation is the lack of information regarding to what extent the drugs were actually ingested. The study reflects Swedish prescription practice and drug availability, and there are certain hypnotic drugs that are approved in other countries but not in Sweden (including eszopiclone, suvorexant, and trazodone, while zaleplon was only approved for use until 2015). Additionally, we did not examine drugs that may be used off‐label for sleep (including quetiapine, mirtazapine, agomelatine, amitriptyline, mianserin) or drugs used both as anxiolytics and sometimes as hypnotics (including benzodiazepines such as oxazepam and diazepam or antihistaminergic drugs). We did not present comorbid diagnoses of insomnia, since they are very rarely recorded in Swedish registers. We also did not study other treatments prescribed for depression. As our study focuses on annual prevalence, we did not take the duration of use for the individual patient into account, nor dosage or quantity of drugs prescribed.

When comparing the rates of hypnotic drug use between patients with depression and the general population using publicly available data from the Swedish National Board of Health and Welfare (all adults aged 20 or older in 2017), we found that the patients with depression in our study had over 3 times higher rates of hypnotic drug use for Z‐drugs (27.6% compared with 8.1%), almost eight times higher for propiomazine (19.7% vs. 2.5%), over 12 times higher for melatonin (12.0% vs. 0.95%), approximately 4 times higher for flunitrazepam (0.6% vs. 0.14%), and around double the rates for nitrazepam (0.3% vs. 0.13%) (The National Board of Health and Welfare, 2023).

A Finnish survey found that 16% of patients with depression used sedatives/hypnotics in the previous year when including patients who did not use mental health services, while 24% of patients in primary care and 32% of patients in specialised care used sedatives/hypnotics (Hämäläinen et al., 2009). The latter group of patients corresponds well to those in our study, where a higher proportion of patients (44%) used hypnotics in the year following diagnosis in 2017.

As expected, in light of studies lending some support to their short‐term use in the treatment of depression (Kishi et al., 2017), Z‐drugs were the most commonly prescribed type of hypnotics. The decrease in the proportion of patients using Z‐drugs, particularly zolpidem, and the substantial reduction in benzodiazepine hypnotic use align with changes seen in the general Nordic populations in recent years (Højlund et al., 2023). Similar declines in zolpidem and benzodiazepine use for sleep have also been seen in the United States (Wong et al., 2020) and France due to stricter regulations (Caillet et al., 2020). Despite limited evidence of its effectiveness in depression (Li, Ma, et al., 2022), our study revealed a nearly 30‐fold increase in melatonin use from 2007 to 2017, likely partly driven by changes in national regulations. In the United States, melatonin use among the general population increased from 0.4% in 1999–2000 to 2.1% in 2017–2018 (Li, Somers, et al., 2022).

Our results indicate similar rates of hypnotic drug use in men and women, despite one study suggesting higher rates of insomnia as a symptom of depression in men than in women (Khan et al., 2002). Hypnotics drug use was higher among older patients and in those with greater comorbidity, aligning with the increased prevalence of insomnia symptoms with higher age (Ohayon, 2002) and comorbidity (Winkelman, 2015) observed in both the general population and patients with depression (Sunderajan et al., 2010).

The higher use of hypnotic drugs observed in severe depression and in patients with previous suicide attempts was expected. Increasing depression severity has been associated with more sleep complaints in adolescents (Urrila et al., 2012). Sleep disturbances and insomnia in particular are risk factors of suicidal thoughts and behaviours (Harris et al., 2020) and previous studies have reported an association between insomnia in depression and suicidality (Agargun et al., 2007; Chellappa & Araújo, 2007). However, hypnotics use has also been associated with an increased risk of suicidality even after adjustments for mental health (McCall et al., 2017; Tubbs et al., 2021). We also found that hypnotics use was far more common in patients diagnosed with depression in an inpatient setting (58.3%), similar to a previous study where 63.6% of inpatients with depression were prescribed hypnotics at discharge (Furihata et al., 2022). Using any hypnotic drug was clearly more common in patients with comorbid non‐alcohol substance use disorder, but for other comorbid psychiatric conditions there were only small differences in overall hypnotics use. This was somewhat surprising considering that anxiety disorders were associated with much more sleep complaints in the STAR*D study (Sunderajan et al., 2010). Still, comorbid conditions did affect the type of substance used. Notably, patients with comorbid ADHD were more likely to use melatonin and slightly less likely to use Z‐drugs compared with all patients with depression. This may be attributed to the perception that melatonin is particularly effective in patients with neuropsychiatric diagnoses, as suggested by studies in children and adolescents (Rzepka‐Migut & Paprocka, 2020).

Our finding that long‐term use of hypnotics was common after a depression diagnosis is of particular interest. Evidence for the effectiveness and safety of long‐term sleep medication is insufficient in chronic insomnia (Medalie & Cifu, 2017; Qaseem, Kansagara, et al., 2016) and hypnotics are typically approved for short‐term use in most European countries (Riemann et al., 2017). The evidence is even more limited for their use in depression (Kishi et al., 2017). Long‐term studies of hypnotics are generally rare, with follow‐up duration not exceeding 12 months in trials (Riemann et al., 2023).

The results of this study mean that in real‐world clinical settings, hypnotic drugs are a common addition to other treatments for depression, suggesting that antidepressants are not perceived as sufficient in many cases. Reflecting increased awareness over the years about the risks associated with benzodiazepines and Z‐drugs, utilisation of such drugs decreased over this period. Instead, melatonin has appeared as an increasingly used alternative.

In conclusion, we found that that hypnotics were used to a large extent in depression in Sweden 2007–2017, even though depression treatment guidelines generally lack recommendations regarding such drugs, making it challenging to evaluate the appropriateness of this use. This seems particularly relevant for long‐term use, which was observed in a considerable proportion of patients up to 3 years after the depression diagnosis. In light of this, the decreasing use of potentially addictive hypnotics during the study period appears beneficial. Considering the results of this study, factors predicting hypnotics use in depression should be further investigated.

## AUTHOR CONTRIBUTIONS

**Adam Nygren:** Writing – original draft; writing – review and editing; project administration; investigation; visualization. **P. Brenner:** Conceptualization; writing – review and editing; data curation; supervision; methodology. **L. Brandt:** Methodology; formal analysis; data curation; visualization; writing – review and editing. **P. Karlsson:** Formal analysis; data curation; methodology; visualization; writing – review and editing. **S. Eloranta:** Writing – review and editing; methodology. **J. Reutfors:** Conceptualization; investigation; writing – review and editing; methodology; project administration; funding acquisition; data curation; supervision; resources.

## CONFLICT OF INTEREST STATEMENT

AN, PB, LB, PK, and JR are affiliated with/employed at the Centre for Pharmacoepidemiology at Karolinska Institutet, which receives grants from several entities (pharmaceutical companies, regulatory authorities, contract research organisations) for the performance of drug safety and drug utilisation studies, and are part of a research collaboration between Karolinska Institutet and Janssen Pharmaceutical NV for which Karolinska Institutet has received/receives grant support. SE declares no conflict of interest.

## Data Availability

The data used for this study may not, according to the ethical permission granted for its use, be shared by the authors to a third party. It is accessible by application to the Swedish authorities (The National Board of Health and Welfare and Statistics Sweden).
